# Comparison of Outcomes of Patients Undergoing Reimplantation versus Bentall Root Procedure

**DOI:** 10.1055/s-0042-1744135

**Published:** 2022-08-07

**Authors:** Lars G. Svensson, Brad F. Rosinski, Nicholas J. Tucker, A. Marc Gillinov, Jeevanantham Rajeswaran, Eric E. Roselli, Douglas R. Johnston, Milind Y. Desai, Brian P. Griffin, Eugene H. Blackstone

**Affiliations:** 1 Department of Thoracic and Cardiovascular Surgery, Heart, Vascular, and Thoracic Institute, Cleveland Clinic, Cleveland, Ohio; 2 The Aorta Center, Heart, Vascular, and Thoracic Institute, Cleveland Clinic, Cleveland, Ohio; 3 Department of Quantitative Health Sciences, Research Institute, Cleveland Clinic, Cleveland, Ohio; 4 Department of Cardiovascular Medicine, Heart, Vascular, and Thoracic Institute, Cleveland Clinic, Cleveland, Ohio

**Keywords:** aortic valve, aortic root, bioprosthesis, surgery

## Abstract

**Background** A bioprosthesis- or mechanical-prosthesis–containing polyester graft (composite graft) is standard surgical management for aortic root aneurysms (Bentall procedure), but particularly in the young patient in whom a bioprosthesis is likely to deteriorate and a mechanical prosthesis mandates life-long anticoagulation, valve-sparing procedures have been devised. One such procedure involves reimplantation of the native aortic valve in the polyester graft. With focus on selecting the optimum procedure for young relatively asymptomatic patients, we compared outcomes of reimplantation of the aortic valve versus the Bentall procedure and identified factors influencing outcomes.

**Methods** From January 2000 to January 2017, 643 adults age ≤ 70 with tricuspid aortic valves underwent elective aortic root replacement with either reimplantation ( *n* = 448/70%) or a composite valve graft (Bentall) procedure ( *n* = 195/30%). Outcomes were compared in 100 propensity-matched pairs.

**Results** Patients with fewer symptoms, less aortic regurgitation (AR), higher left ventricular ejection fraction, and smaller cross-sectional aortic area/height ratio had a higher likelihood of valve repair with reimplantation (all *p* < 0.02) versus receiving a Bentall procedure. Operative mortality was 0.16% (reimplantation, 1/448, 0.22%; Bentall 0/195, 0%). After reimplantation, 8-year freedom from severe AR was 95% and 10-year freedom from reintervention was 98%. Ten-year survival was 95%. Higher preoperative AR grade ( *p* < 0.0001) but not larger root diameter ( *p* = 0.3) was associated with higher grade of late regurgitation after a reimplantation procedure. Among propensity-matched patients, reimplantation compared with a Bentall was associated with similar 10-year survival (89% vs. 94%), but more late AR (8-year freedom from severe AR: 93% vs. 99.9%) and greater early reduction in, but similar late, left ventricular mass (104 vs. 105 g•m ^–2^ at 8 years).

**Conclusion** Excellent aortic valve reimplantation results versus Bentall lead us to recommend reimplantation more often in patients who present with even moderately severe or severe AR and significantly enlarged aortic roots.

## Introduction

There is general agreement regarding performing surgery on operable patients with severe aortic regurgitation accompanying aortic root aneurysms > 5.0 to 5.5 cm, 1 2 a large left ventricle, and left ventricular dysfunction. Standard surgical management is a Bentall procedure in which either a bioprosthesis or a mechanical prosthesis is incorporated within a polyester graft (composite graft).

Although the Bentall procedure is associated with low mortality and morbidity, bioprostheses deteriorate, more rapidly in the younger patient, requiring reoperation, and mechanical prostheses require life-long anticoagulation with its risks. The alternative is an aortic valve-sparing operation. We and others have shown that it is possible to achieve successful aortic root repair in more than 95% of cases—with less than 1% mortality and 92 to 98% freedom from aortic valve reoperation at 5 years 3 4 5 6 7 8 —using the valve-sparing reimplantation operation first described by David for aortic root aneurysms with or without aortic valve regurgitation and with improved subsequent modifications. 3 4 5 6 7 8 9 10 However, there are few studies evaluating outcomes following a reimplantation procedure compared with the Bentall procedure at 8 to 10 years. 3 4 5 6 7 8 9 10

Therefore, we evaluated early and late outcomes of a reimplantation procedure compared with a Bentall procedure and how surgery, either early or late in the disease process, influenced success.

## Materials and Methods

### Patients

From January 2000 to January 2017, 1,159 patients underwent aortic valve reimplantation with aortic root replacement or Bentall operation at Cleveland Clinic. Of these, 516 were excluded for age > 70 years or < 18 years, aortic valve reoperation, aortic valve stenosis, infective endocarditis, aortic dissection, emergency surgery, or abnormal cusp number (unicuspid, bicuspid, and quadricuspid). Of the remaining 643 patients, 448 had an aortic valve reimplantation procedure, and 195 underwent a Bentall operation that incorporated either a bioprosthesis ( *n* = 147, 75%) or a mechanical prosthesis ( *n* = 48, 25%). Mean age at operation was 48 ± 13 years and 58 ± 10 years in the reimplantation and Bentall groups, respectively, with male predominance ( Table 1 ).

**Table 1:** Preoperative patient characteristics

Characteristics	Unmatched cohorts					Propensity-matched cohorts				
	Reimplantation ( *n* = 448)		Bentall ( *n* = 195)		Std. Diff. (%)	Reimplantation ( *n* = 100)		Bentall ( *n* = 100)		Std. Diff. (%)
	*n* a	No. (%) or mean ± SD	*n* a	No. (%) or mean ± SD		*n* a	No. (%) or mean ± SD	*n* a	No. (%) or mean ± SD	
*Demographics* :										
Age (y)	448	48 ± 13	195	58 ± 10	88	100	58 ± 8.0	100	56 ± 10	–12
Female	448	85 (19)	195	21 (11)	–23	100	12 (12)	100	13 (13)	3.0
Height (cm)	448	182 ± 10	195	179 ± 10	–22	100	181 ± 9.0	100	179 ± 11	–16
BMI (kg•m ^–2^ )	444	27 ± 5.1	192	29 ± 5.9	37	99	29 ± 5.2	98	29 ± 6.0	–5.8
*NYHA functional class* :	414		179		33	91		87		7.9
I		263 (64)		82 (46)			42 (46)		37 (43)	
II		129 (31)		83 (46)			42 (46)		42 (48)	
III		21 (5.1)		14 (7.8)			7 (7.7)		8 (9.2)	
IV		1 (0.24)		0 (0)			0 (0)		0 (0)	
*Aortic dimensions*^b^ :										
Root diameter (cm)	448	5.0 ± 0.52	195	5.2 ± 0.74	33	100	5.1 ± 0.61	100	5.1 ± 0.65	–9.5
Root area/height ratio	448	11 ± 2.3	195	12 ± 3.5	41	100	12 ± 2.8	100	12 ± 2.9	–4.2
Ascending diameter (cm)	445	4.5 ± 0.90	194	4.9 ± 1.0	39	99	4.8 ± 0.86	99	4.8 ± 0.92	–4.5
*Aortic valve pathology* :										
AR grade	448		195		114	100		100		14
None		186 (42)		14 (7.2)			13 (13)		13 (13)	
Mild		42 (9.4)		6 (3.1)			4 (4.0)		3 (3.0)	
Moderate		97 (22)		39 (20)			24 (24)		20 (20)	
Severe		123 (27)		136 (70)			59 (59)		64 (64)	
*Mitral valve regurgitation* :	444		192		–4.3	98		98		–2.8
None		349 (79)		147 (77)			74 (76)		73 (74)	
Mild		46 (10)		26 (14)			10 (10)		13 (13)	
Moderate		20 (4.5)		12 (6.3)			5 (5.1)		5 (5.1)	
Severe		29 (6.5)		7 (3.7)			9 (9.2)		7 (7.2)	
*Ventricular morphology and function* :										
LV mass index (g•m ^−2^ )	402	116 ± 42	178	142 ± 47	57	87	136 ± 52	93	139 ± 51	7.5
Ejection fraction (%)	439	57 ± 5.8	191	54 ± 7.4	–44	98	55 ± 6.0	99	55 ± 7.0	3.4
*Cardiac rhythm* :										
AF or flutter	440	24 (5.5)	191	19 (9.9)	17	98	8 (8.2)	97	9 (9.3)	4.0
*Coronary artery stenosis* :										
Left circumflex artery ≥ 50%	446	15 (3.4)	190	18 (9.5)	25	99	9 (9.1)	97	5 (5.2)	–15
LAD ≥ 50%	447	27 (6)	193	32 (17)	34	100	16 (16)	100	13 (13)	–8.5
Right coronary artery ≥ 50%	448	15 (3.3)	191	21 (11)	30	100	9 (9)	98	8 (8.2)	–3.0
Left main disease ≥ 50%	446	0 (0)	190	3 (1.6)	18	99	0 (0)	100	2 (2)	20
Preoperative ICD	448	1 (0.22)	195	0 (0)	—	—	—	—	—	—
Preoperative pacemaker	448	1 (0.22)	195	1 (0.51)	—	—	—	—	—	—
*Noncardiac comorbidities* :										
Hypertension	447	309 (69)	195	156 (80)	25	100	80 (80)	100	81 (81)	2.5
Peripheral arterial disease	448	24 (5.4)	195	14 (7.2)	7.5	100	8 (8.0)	100	5 (5.0)	–12
Pharmacologically treated diabetes	445	15 (3.4)	195	15 (7.7)	19	100	6 (6.0)	100	5 (5.0)	–4.4
COPD	448	52 (12)	195	21 (11)	–2.7	100	12 (12)	100	12 (12)	0.0
History of smoking	448	180 (40)	195	96 (49)	18	100	54 (54)	100	50 (50)	–8.0
Creatinine (mg•dL ^–1^ )	446	0.95 ± 0.21	194	1.04 ± 0.48	24	100	0.98 ± 0.25	99	1.0 ± 0.62	14

Abbreviations: AF, atrial fibrillation; AR, aortic regurgitation; BMI, body mass index; COPD, chronic obstructive pulmonary disease; ICD, implantable cardioverter-defibrillator; LAD, left anterior descending coronary artery; LV, left ventricle; NYHA, New York Heart Association; SD, standard deviation; Std Diff, standardized difference (Bentall-Reimplantation).

a Patients with data available.

b Echocardiographic.

Operative techniques included previously described modifications 4 11 of David's reimplantation method, which entails mobilizing the aortic valve, reimplanting it into a polyester tube graft, attaching coronary buttons, and replacing the aneurysmal aorta, 3 8 9 10 12 including plegeted sutures in the left ventricular outflow tract and use of a Hegar dilator. Concomitant mitral valve surgery was performed in 35 patients (7.8%), coronary artery bypass grafting in 31 (6.9%), and 34 (7.6%) required circulatory arrest ( Table 2 ).

**Table 2:** Operative details

Detail	Unmatched cohorts					Propensity-matched cohorts				
	Reimplantation ( *n* = 448)		Bentall ( *n* = 195)		Std. Diff. (%)	Reimplantation ( *n* = 100)		Bentall ( *n* = 100)		Std. Diff. (%)
	*n* a	No. (%) or mean ± SD	*n* a	No. (%) or mean ± SD		*n* a	No. (%) or mean ± SD	*n* a	No. (%) or mean ± SD	
*Concomitant procedures* :										
Descending aorta grafting	448	5 (1.1)	195	1 (0.51)	–6.7	100	0 (0)	100	1 (1.0)	14
CABG	448	31 (6.9)	195	43 (22)	44	100	19 (19)	100	14 (14)	–13
Ablation procedure for AF	448	21 (4.7)	195	21 (11)	23	100	8 (8.0)	100	9 (9.0)	3.6
Mitral valve repair	448	34 (7.6)	195	9 (4.6)	–12	100	9 (9.0)	100	6 (6.0)	–11
Mitral valve replacement	448	1 (0.22)	195	3 (1.5)	14	100	1 (1.0)	100	3 (3.0)	14
Tricuspid valve repair	448	7 (1.6)	195	1 (0.51)	–10	100	2 (2.0)	100	1 (1.0)	–8.2
*Support* :										
Circulatory arrest	448	34 (7.6)	195	27 (14)		100	9 (9)	100	14 (14)	
Circulatory arrest time (min)	34	18 ± 13	27	16 ± 7.4		9	12 ± 3.1	14	18 ± 7.7	
Myocardial ischemic time (min)	448	106 ± 37	195	95 ± 36		100	114 ± 34	100	96 ± 36	
CPB time (min)	448	127 ± 43	195	117 ± 45		100	136 ± 40	100	116 ± 44	

Abbreviations: AF, atrial fibrillation; CABG, coronary artery bypass grafting; CPB, cardiopulmonary bypass; SD, standard deviation; Std Diff, standardized difference (Bentall-Reimplantation).

a Patients with data available.

### Data

Patient characteristics and operative details were abstracted prospectively into the Cleveland Clinic Thoracic Aorta Database, data that are approved for use in research by the institutional review board, with patient consent waived.

### Endpoints

Endpoints were (1) postoperative in-hospital mortality and adverse events defined by the Society of Thoracic Surgeons national database ( https://www.sts.org/registries-research-center/sts-national-database ), (2) longitudinal postoperative aortic valve regurgitation and stenosis and left ventricular reverse remodeling, (3) reoperation on the aortic valve or thoracic aorta, and (4) time-related mortality.

Transthoracic echocardiography was used to assess postoperative aortic valve regurgitation, mean aortic valve gradient, and left ventricular mass index. 13 Echocardiography was performed routinely before index hospital discharge and at referring physician discretion during follow-up. A total of 1,759 echocardiograms were available for 591 patients (92% of the study cohort) ( Supplementary Fig. S1 ). No data in this report are based on intraoperative transesophageal echocardiography, which was routinely performed. Interpretation of follow-up echocardiograms was obtained at as many time points as available for each patient. Echocardiographic data were censored at time of aortic valve or thoracic aorta reintervention, death, or final follow-up.

Systematic follow-up performed at 2, 5, 10, 15, and 20 years was used to identify aortic valve reinterventions. In the reimplantation group, 50% were followed > 10.5 months, 25% > 4.2 years, and 5% > 11 years; in the Bentall group, 50% were followed > 3.6 months, 25% > 4.5 years, and 5% > 12 years.

Adding supplemental vital status information obtained before November 2011 (Social Security Death Master File) 14 to systematic follow-up, in the reimplantation group 50% of patients were followed > 2.8 years, 25% > 6 years, and 10% > 9 years, and in the Bentall group 50% of patients were followed > 2.4 years, 25% > 7 years, and 10% > 11 years.

### Data Analysis

SAS statistical software (SAS version 9.2; SAS Institute, Cary, NC) and R version 3.3.1 were used for analysis. Continuous variables are summarized as mean ± standard deviation or as 15th/50th (median)/85th percentiles when values are skewed; comparisons are based on the Wilcoxon rank-sum nonparametric test. Categorical data are summarized using frequencies and percentages; comparisons are based on the chi-squared test or Fisher's exact test. Uncertainty is expressed by confidence limits equivalent to ± 1 standard error (68%).

#### Variables associated with Reimplantation versus a Bentall Procedure

A parsimonious model for reimplantation versus a Bentall procedure was developed using logistic regression, with variable selection from preoperative patient variables and intended concomitant procedures listed in Supplementary Appendix S1 . Variable selection, with *p* -value criterion for retention of variables in the model of 0.05, used bootstrap bagging with 500 bootstrap data sets. 15 Frequency of occurrence of variables related to reimplantation versus Bentall procedure was ascertained (aggregation step) and indicated the reliability of each variable. Variables with bootstrap reliability ≥ 50% were retained in the final model.

Prior to multivariable analysis, we employed fivefold multiple imputation 16 using a Markov chain Monte Carlo technique.

For complementary analysis, random forest classification 17 was performed to assess possible nonlinear relationships between likelihood of reimplantation and continuous patient characteristics, using risk-adjusted partial-dependency plots. 18 All variables listed in Supplementary Appendix S1 were included in the analysis, without variable selection. Owing to a strong correlation among different expressions of aortic root size, we performed separate analyses with (1) aortic root diameter, (2) root area/height ratio, and (3) both root diameter and area/height ratio. Missing data were imputed using “on the fly” random forest imputation. 19

#### Longitudinal Data Analysis

Postoperative and follow-up transthoracic echocardiograms were analyzed for temporal pattern of change using a nonlinear multiphase mixed-effects cumulative logistic regression model for longitudinal ordinal data and a nonlinear mixed-effects regression model for continuous data, both with patient as the random effect. 20

#### Time-Related Events

Freedom from aortic valve reintervention and survival were assessed nonparametrically by the Kaplan–Meier estimator and time-varying instantaneous risk of death by a multiphase parametric hazard model. 21

#### Development and Use of Propensity Score

Because patient characteristics differed for reimplantation versus a Bentall procedure, propensity-matched cohorts were compared using the parsimonious model of variables associated with reimplantation, other patient demographic variables, symptoms, cardiac and noncardiac comorbidities, and procedure variables that might be related to unrecorded selection factors (41 variables, Supplementary Table S1 , *c* -statistic = 0.91; Supplementary Appendix S1 ). A propensity score was calculated for each patient by solving the model for the probability of being in the reimplantation group and used to match Bentall cases 1:1 by greedy matching. Bentall cases whose propensity scores deviated > 0.10 from those of reimplantation cases were considered unmatched. This process yielded 100 well-matched pairs (51% of possible matches; see Table 1 and Supplementary Figs. S2A and S2B ).

## Results

### Selection of Reimplantation versus a Bentall Procedure

Patients were more likely to undergo reimplantation than a Bentall procedure ( Supplementary Fig. S3 , and see Table 1 ) if they were asymptomatic (64% vs. 46%, *p* = 0.0008), younger than age 50 years ( Supplementary Fig. S4A ), had less preoperative aortic regurgitation, better left ventricular function ( Supplementary Fig. S4B ), smaller aortic root ( Supplementary Fig. S4C ) or smaller aortic root/height ratio ( Supplementary Fig. S4D ), and were operated on more recently ( Supplementary Fig. S4E ).

### Safety of Reimplantation versus a Bentall Procedure

Hospital mortality was 0.22% (1/448) after reimplantation and 0% (0/195) after a Bentall procedure ( Table 3 ). Among propensity-matched patients, occurrence of in-hospital adverse events, including stroke, renal failure requiring dialysis, reoperation for postoperative bleeding, and postoperative atrial fibrillation, was similar between groups ( Table 3 ). However, the reimplantation group had a median postoperative length of stay that was 1 day shorter.

**Table 3:** In-hospital outcomes

Outcome	Unmatched cohorts				Propensity-matched cohorts				
	Reimplantation ( *n* = 448)		Bentall ( *n* = 195)		Reimplantation ( *n* = 100)		Bentall ( *n* = 100)		*p* -Value
	*n* a	No. (%) or 15th/50th/85th percentiles	*n* a	No. (%) or 15th/50th/85th percentiles	*n* a	No. (%) or 15th/50th/85th percentiles	*n* a	No. (%) or 15th/50th/85th percentiles	
Hospital death	448	1 (0.22)	195	0 (0)	100	0 (0)	100	0 (0)	—
Stroke	448	1 (0.22)	195	1 (0.51)	100	1 (1.0)	100	0 (0)	> 0.9
Reoperation for valve dysfunction	448	1 (0.22)	195	0 (0)	100	1 (1.0)	100	0 (0)	> 0.9
Reoperation for bleeding	448	8 (1.8)	195	8 (4.1)	100	1 (1.0)	100	4 (4.0)	0.4
Renal failure requiring dialysis	445	1 (0.22)	194	1 (0.52)	100	0 (0)	99	1 (1.0)	0.5
Prolonged ventilation (> 24 h)	447	19 (4.3)	181	13 (7.2)	100	2 (2.0)	97	7 (7.2)	0.1
New-onset atrial fibrillation	417	104 (25)	172	77 (45)	90	36 (40)	88	41 (47)	0.4
Postoperative ICD	447	6 (1.3)	195	3 (1.5)	100	3 (3)	100	0 (0)	> 0.9
Postoperative pacemaker	447	8 (1.8)	194	13 (6.7)	100	2 (2)	100	6 (6)	0.001
*Transfusions* :									
Red blood cell	448	113 (25)	195	58 (30)	100	18 (18)	100	32 (32)	0.02
Platelets	409	130 (32)	164	65 (40)	84	33 (39)	91	37 (41)	0.8
Fresh frozen plasma	448	93 (21)	195	44 (23)	100	26 (26)	100	26 (26)	> 0.9
Any transfused product	448	191 (43)	195	93 (48)	100	44 (44)	100	51 (51)	0.3
*Length of stay* :	448		195		100		100		
Intensive care unit (h)		22/28/73		23/46/96		22/27/98		23/47/97	0.08
Postoperative (d)		5.0/6.1/9.0		5.2/7.2/11		5.1/6.1/10		5.2/7.1/12	0.01

Abbreviation: ICD, implantable cardioverter-defibrillator.

a Patients with data available.

### Effectiveness of Valve Reimplantation

**Fig. 1:**
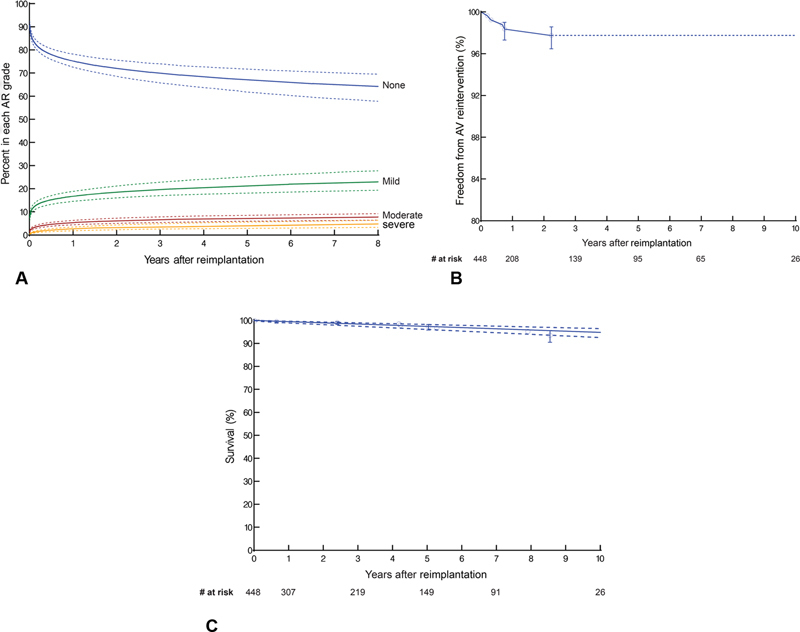
Outcomes after a reimplantation procedure. ( **A** ) Postoperative prevalence of severe aortic regurgitation (AR). Solid lines represent the longitudinal trend in each grade, and symbols represent grouped data without regard to repeated measures to provide crude verification of model fit. ( **B** ) Freedom from aortic valve reintervention. Each symbol represents a reintervention and vertical lines 68% confidence limits equivalent to ± 1 standard error. Numbers below horizontal axis represent patients still being followed. ( **C** ) Survival. Each symbol represents a death and vertical bars 68% confidence limits equivalent to ± 1 standard error. Solid line within a 68% confidence band represents parametric estimates. Numbers below horizontal axis represent patients still being followed.

**Fig. 2:**
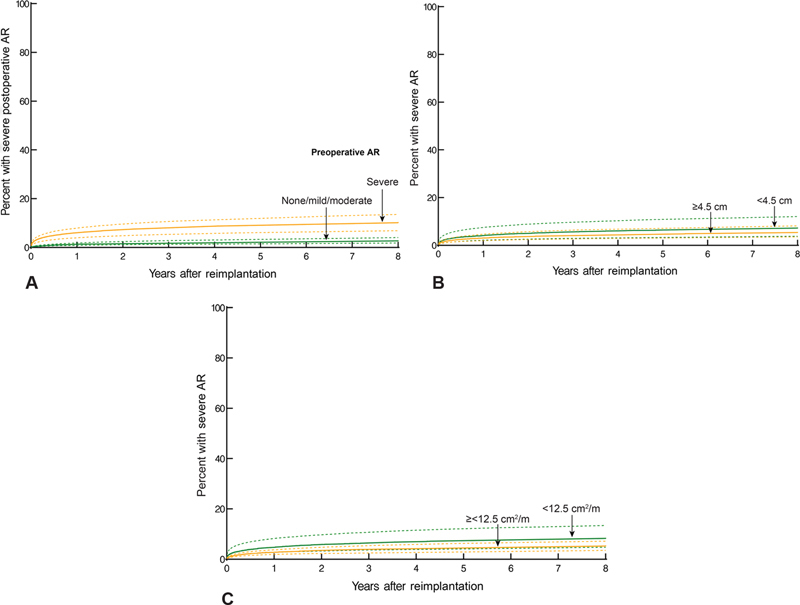
Relationship of severe postoperative aortic regurgitation (AR) to preoperative variables. Format is as in Fig. 1A . ( **A** ) Greater preoperative severity of AR was associated with more severe late AR. ( **B** ) Preoperative aortic root diameter was not associated with severe postoperative AR. ( **C** ) Preoperative aortic root area/height ratio was not associated with severe postoperative AR.

Among all 448 patients who underwent aortic valve reimplantation, freedom from severe aortic regurgitation was 96% at 5 years and 95% at 8 years ( Fig. 1A ), 10-year freedom from aortic valve reintervention was 98% with 5 patients undergoing aortic valve reintervention, all for aortic regurgitation secondary to cusp dysfunction ( Fig. 1B ), and 5- and 10-year survival was 97 and 95% ( Fig. 1C ). Higher likelihood of postoperative aortic regurgitation was associated with higher preoperative grade of aortic regurgitation ( Fig. 2A ; *p* < 0.0001); however, neither preoperative aortic root diameter ( Fig. 2B ; *p* = 0.3) nor area/height ratio ( Fig. 2C ; *p* = 0.14) was associated with greater regurgitation. Mean gradient was 7 mm Hg at 5 and 8 years after surgery ( Supplementary Fig. S5 ), and left ventricular mass, 120 g·m ^–2^ at time of surgery was 104 g·m ^–2^ at 5 years and 105 g·m ^–2^ at 8 years after surgery ( Supplementary Fig. S6 ). In patients who had both aortic valve reimplantation and mitral valve repair, freedom from reoperation was 100% at 10 years, not significantly different from that of patients having reimplantation alone ( *p* = 0.5).

**Fig. 3:**
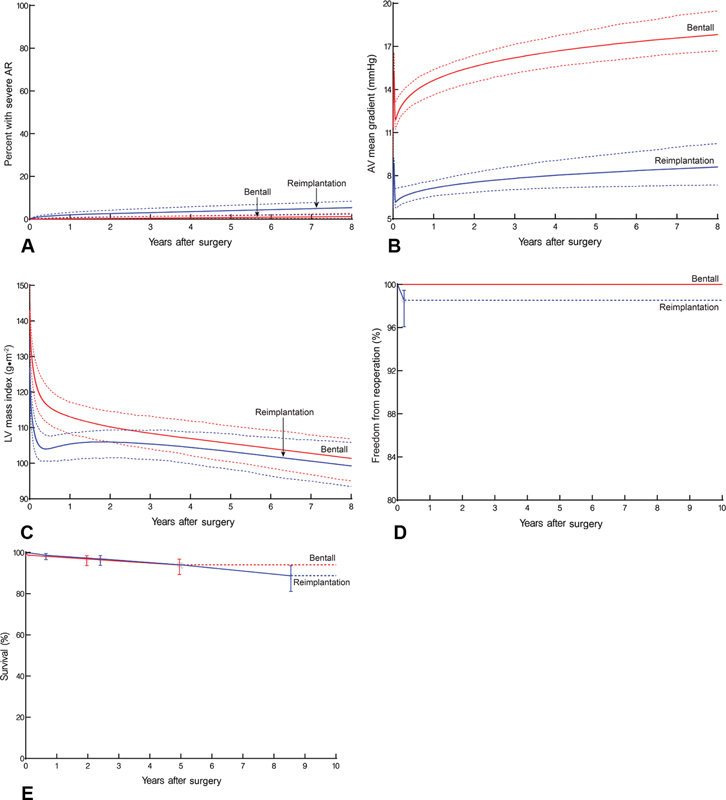
Outcomes in propensity-matched cohorts of patients after undergoing reimplantation versus a Bentall procedure. ( **A** ) Aortic regurgitation (AR). Solid lines represent parametric estimates of percentage of patients (mean effect) with severe AR after surgery. Symbols represent data grouped (without regard to repeated measurements) within time frames to provide a crude verification of model fit. ( **B** ) Temporal trend of aortic valve (AV) mean gradient. Symbols represent data grouped (without regard to repeated measurements) within time frames to provide a crude verification of model fit. ( **C** ) Left ventricular (LV) mass index. Format is as in panel **B** . ( **D** ) Freedom from reoperation on the aortic valve. Solid lines represent parametric estimates. Symbols are nonparametric Kaplan–Meier estimates, and vertical bars represent 68% confidence limits. ( **E** ) Survival. Format is as in panel **D** .

### Outcomes of Reimplantation versus Bentall Procedure

The propensity-matched reimplantation group, which consisted of patients with Bentall-like preoperative characteristics ( Supplementary Table S1 ), demonstrated significantly more severe postoperative aortic regurgitation than the Bentall group; at 8 years, prevalence was 7.2% compared with 0% in the Bentall group ( *p* = 0.02; Fig. 3A ). The reimplantation group experienced a lower mean gradient ( Fig. 3B ) and a rapid early decrease in mass index ( Fig. 3C ) compared with the Bentall group ( *p =* 0.04 and *p* = 0.007, respectively), although as time progressed, the difference narrowed ( *p* = 0.8 for the late phase). At 10 years, there was no statistically significant difference in freedom from reintervention on the aortic valve (reimplantation, 98%; Bentall, 100%, *p* = 0.8; Fig. 3D ). There was also no significant difference in survival between the matched reimplantation and Bentall groups at 10 years ( *p* = 0.8; Fig. 3E ).

## Discussion

### Principal Findings

The current study shows a high level of repair success (> 95%) and low mortality for patients undergoing reimplantation for aortic root aneurysms with or without aortic regurgitation. Specifically, mortality was 0.22%, repair success high, and freedom from reintervention 98% at 10 years. This study discerned potential benefits of early intervention with valve reimplantation before a large root developed with potential tearing of cusps, more severe aortic regurgitation, or symptoms.

### Reimplantation versus Bentall Procedure

The Bentall group of patients experienced excellent outcomes; however, patients undergoing reimplantation with more advanced disease were more likely to experience severe late aortic regurgitation compared with those undergoing a Bentall procedure, despite the negligible difference in valve reintervention and survival.

Clearly, for aortic root reimplantation, high mortality would negate the benefit of preventing aortic dissection or rupture, heart failure, and late death; however, in this study we have shown that the operation is as safe as the Bentall procedure. Importantly, reimplantation circumvents potential for prosthetic degeneration of bioprosthetic composite grafts and the need for anticoagulation therapy for mechanical composite grafts.

The decision to attempt and the subsequent success at completing a valve reimplantation was influenced by several factors. More symptomatic patients and patients with worse left ventricular dysfunction and more severe aortic regurgitation had lower success, but larger aortic root size did not influence reimplantation success, although a smaller cross-sectional area/height ratio was associated with greater likelihood of successful reimplantation. We inform patients who have aortic root aneurysms with tricuspid valves and moderate or less aortic valve regurgitation that they will have a 90 to 95% chance of valve preservation, and if less than mild regurgitation, a greater than 95% chance. Indeed, this study confirms high success in this group of patients. In an earlier paper, we reported that only 2% of patients had an increase in aortic regurgitation after surgery. 11

We believe that the reason for less success with more severe regurgitation is the greater likelihood of more severe lacerations, perforations, torn cusps, stretched cusps, and prolapse of more than one cusp, making a long-term durable repair more difficult to achieve, and an additional argument for earlier surgery. 22

### Long-Term Durability

We compared outcomes of reimplantation with those of a propensity-matched population, which showed no early or late increased risk of mortality and without risk of late failure of biological Bentalls. 12 Previously, we found that late risk for new dissection after reimplantation was only 1.4%, and most were in the descending aorta and associated with a connective tissue disorder. 8 If patients with aortic cross-sectional area-to-height ratio > 10 are not operated on, late survival is reduced partly because the risk of dissection increases without surgery. 23

### Clinical Implications

Based on this study, > 95% freedom from reoperation and severe regurgitation at 10 years can be expected for tricuspid aortic valves with reimplantation. Critical to the success of aortic valve repairs is addressing the CLASS schema factors (Commissures, Leaflets, Anulus, Sinutubular junction, and Sinuses) that contribute to aortic valve competence 8 11 ; the reimplantation procedure also braces the root and aortic valve, which is analogous to use of anuloplasty for mitral valve repair. 24 Use of commissure figure-of-8 sutures to repair cusp prolapse during reimplantation improves valve repair success.

We believe the modifications we use with pledgets in the left ventricular outflow tract, use of a 30-mm tube graft in most patients, and reducing anular size based on body surface area using Hegar dilators results in a reproducible and reliable procedure with good late valve function. 4 11

### Limitations

This is a single-institution observational study comparing reimplantation with Bentall composite valve grafts. We did not have a control arm of medically treated patients with enlarged aortas and aortic regurgitation. There are, however, reports of medically treated patients with enlarged aortas, but with associated risk of dissection or rupture; most of these patients transition to surgery. 25

## Conclusion

This study demonstrates that reimplantation or root replacement with a Bentall type procedure, with or without aortic regurgitation, can be performed with excellent early and late outcomes, leading us to recommend reimplantation more often in patients who present with even moderately severe or severe aortic regurgitation and significantly enlarged aortic roots. A key to success is learning a reproducible procedure by the methods we use.

## Editor's Commentary

*Tirone E. David, MD, FRCSC*

*Division of Cardiac Surgery, Toronto General Hospital, University of Toronto, Toronto, Ontario, Canada*

This study compared the outcomes of reimplantation of the aortic valve with Bentall procedure with mechanical as well as bioprosthetic valves. Although the sample size is relatively large (particularly for the reimplantation group) the proportion of patients followed beyond 1 year was very small (50% of reimplantation was followed > 10.5 months and 50% of Bentall > 3.6 months). Thus, interpretation of outcomes at 10 years should be made with caution. In addition, patients with mechanical valves likely had valve-related complications such as bleeding and stroke and were not accounted for. Patients with bioprosthetic valve did not have any valve failure because of short follow-up. Based on the data presented the authors should have concluded that these two operations provide similar result up to 10 years and reimplantation was not better than Bentall.
