# Supplementary material for: Comparison of Outcomes of Patients Undergoing Reimplantation versus Bentall Root Procedure
Source: Aorta (Stamford). 2022 Aug 7;10(2):57–68. doi: 10.1055/s-0042-1744135 (PMC9357471; doi:10.1055/s-0042-1744135)
Supplement: Supplementary file 1 — Supplementary Material [file 10-1055-s-0042-1744135-s200057.pdf]

Supplementary Table S1 Variables associated with higher likelihood of reimplantation versus Bentall procedure

Variables	Coefficient ± SE	*p*	Reliability (%)[a]
Younger age[b]	–2.4 ± 0.34	< 0.0001	100
Lower grade of AR	–0.81 ± 0.097	< 0.0001	100
Smaller aortic root area/height ratio[c]	–0.32 ± 0.13	0.02	63
Higher LVEF[d]	1.2 ± 0.41	0.003	68
Lower creatinine clearance[e]	–0.15 ± 0.048	0.002	71
No concomitant CABG	1.2 ± 0.32	0.0003	64
More recent operation[f]	3.1 ± 0.64	< 0.0001	79

Abbreviations: AR, aortic regurgitation; CABG, coronary artery bypass grafting; LVEF, left ventricular ejection fraction; SE, standard error.
[a]Percent of times variable appeared in 500 bootstrap models.
[b]$(Age/50)^2$, squared transformation; see ►**Supplementary Fig. S4A**.
[c]$(\text{Aortic root area/height ratio}/10)^2$, squared transformation; see ►**Supplementary Fig. S4D**.
[d]$(LVEF/50)^2$, squared transformation; see ►**Supplementary Fig. S4B**.
[e]$(\text{Creatinine clearance}/60)^2$, squared transformation.
[f]Log (interval from date of operation to 1/1/2000), logarithmic transformation; see ►**Supplementary Fig. S4E**.

 © 2022. The Author(s).

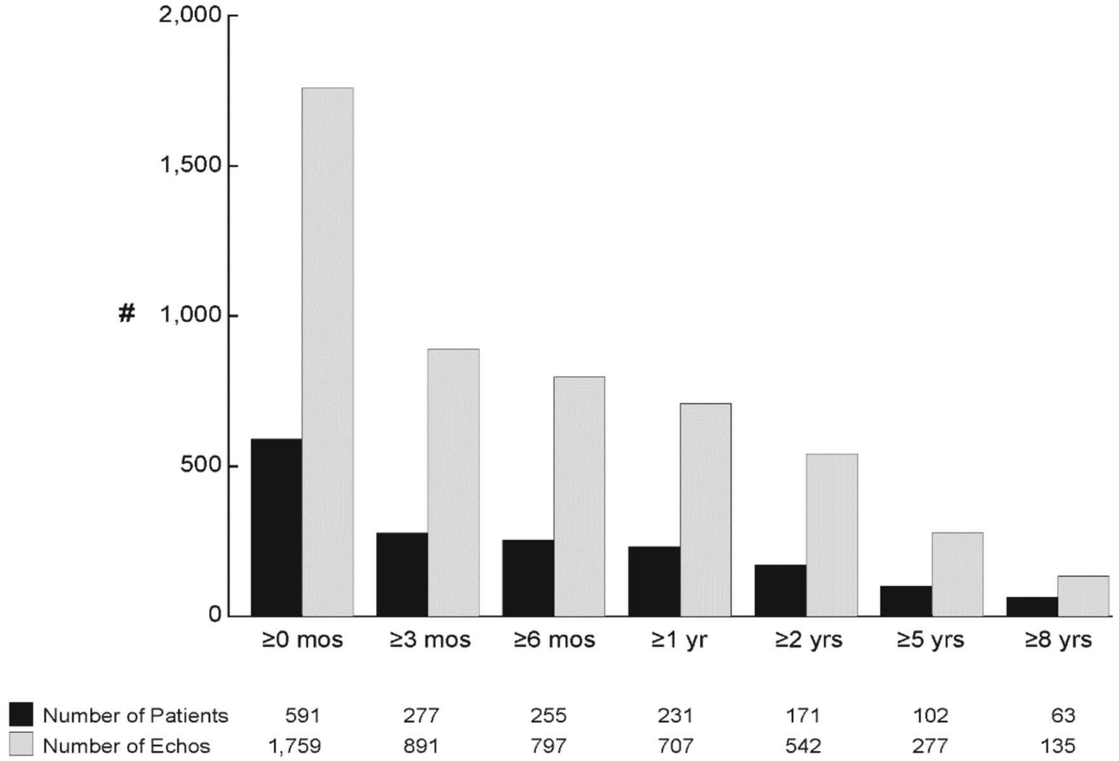

Supplemental Fig. S1 Patients and echocardiograms (echos) available for analysis at particular time points during follow-up.

 © 2022. The Author(s).

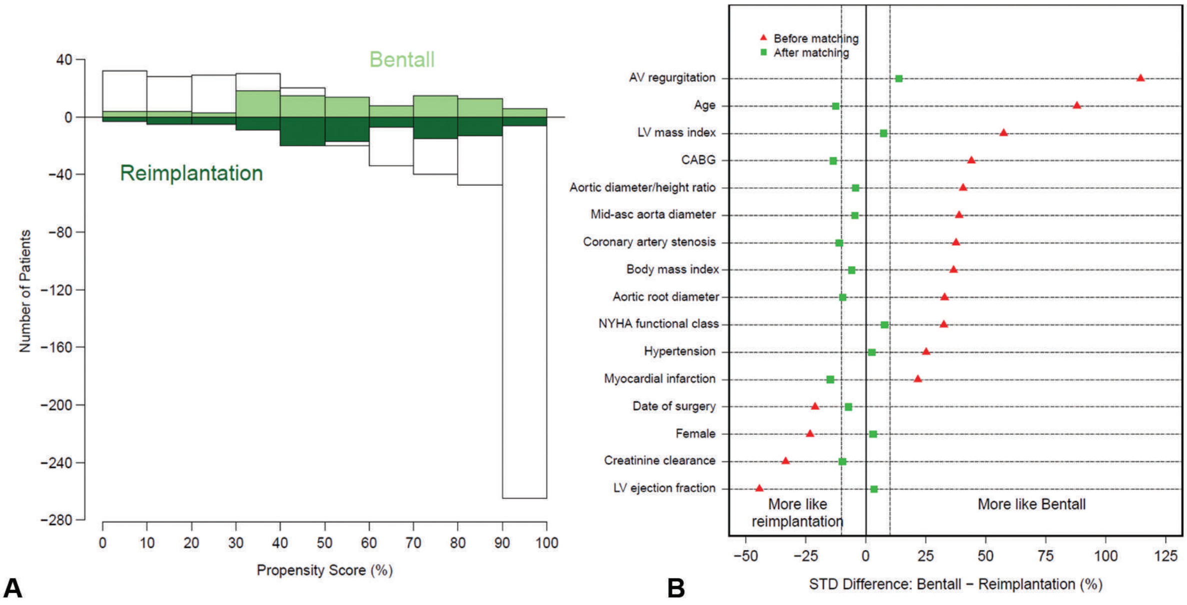

Supplementary Fig. S2 Quality of propensity matching of patients undergoing reimplantation versus a Bentall procedure. (**A**) Mirrored histogram of distribution of propensity scores for both groups. Shaded areas represent matched patients. (**B**) Standardized differences of selected variables before and after matching. (Austin PC, Mamdani MM. A comparison of propensity score methods: a case-study estimating the effectiveness of post-MI statin use. *Stat Med.* 2006;25:2084–106.) AV, aortic valve; LV, left ventricular; CABG, coronary artery bypass grafting; mid-asc, mid-ascending; NYHA, New York Heart Association; STD, standardized.

 © 2022. The Author(s).

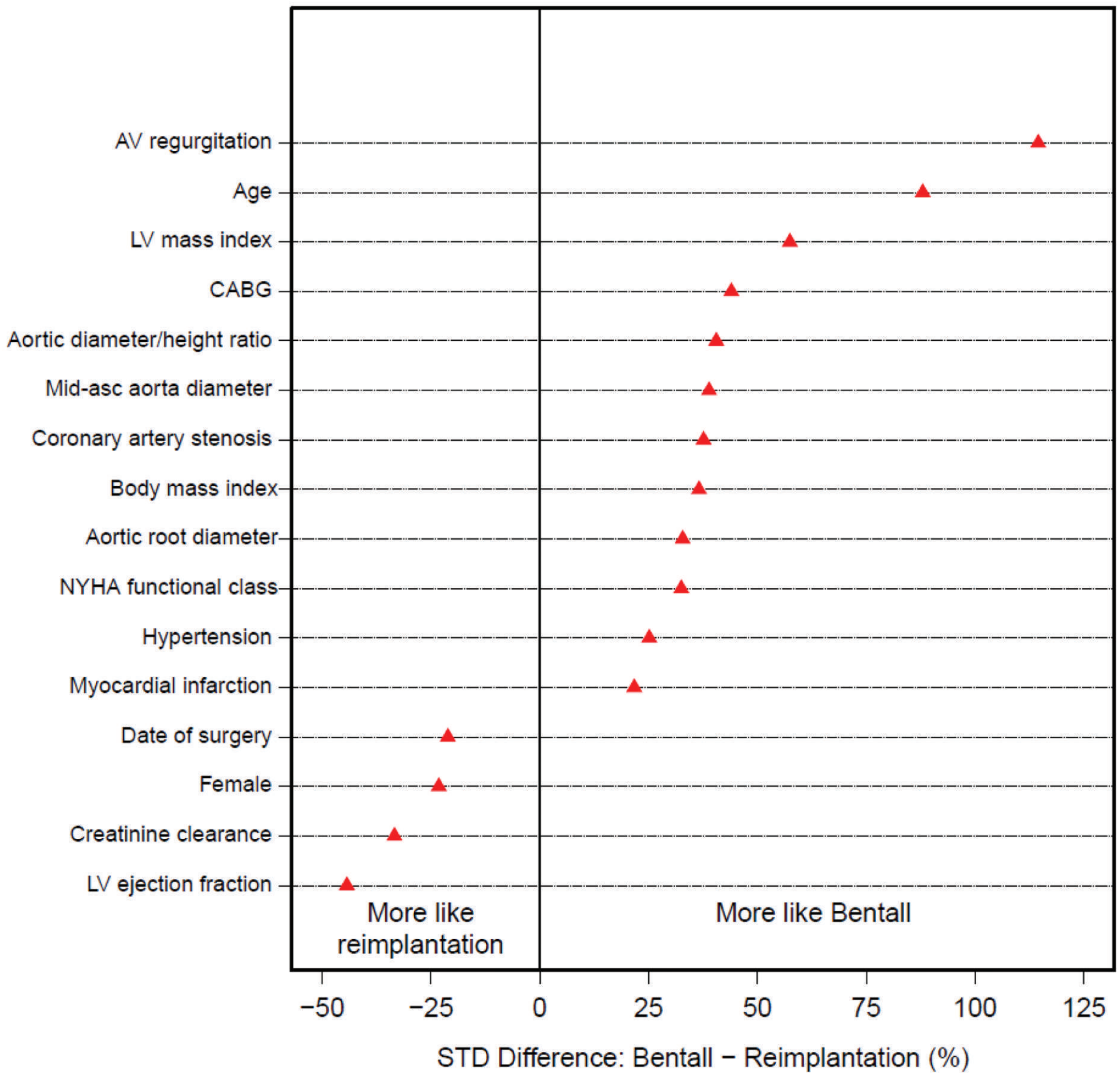

Supplementary Fig. S3 Standardized differences for selected variables between patients undergoing a reimplantation procedure for aortic root aneurysm and those undergoing a Bentall operation (polyester conduit containing a mechanical aortic valve prosthesis or bioprosthesis). To the left of the vertical line are characteristics more like those in patients undergoing a reimplantation and to the right those in patients undergoing a Bentall operation. AV, aortic valve; LV, left ventricle; CABG, coronary artery bypass grafting; mid-asc, mid-ascending; NYHA, New York Heart Association; STD, standardized.

 © 2022. The Author(s).

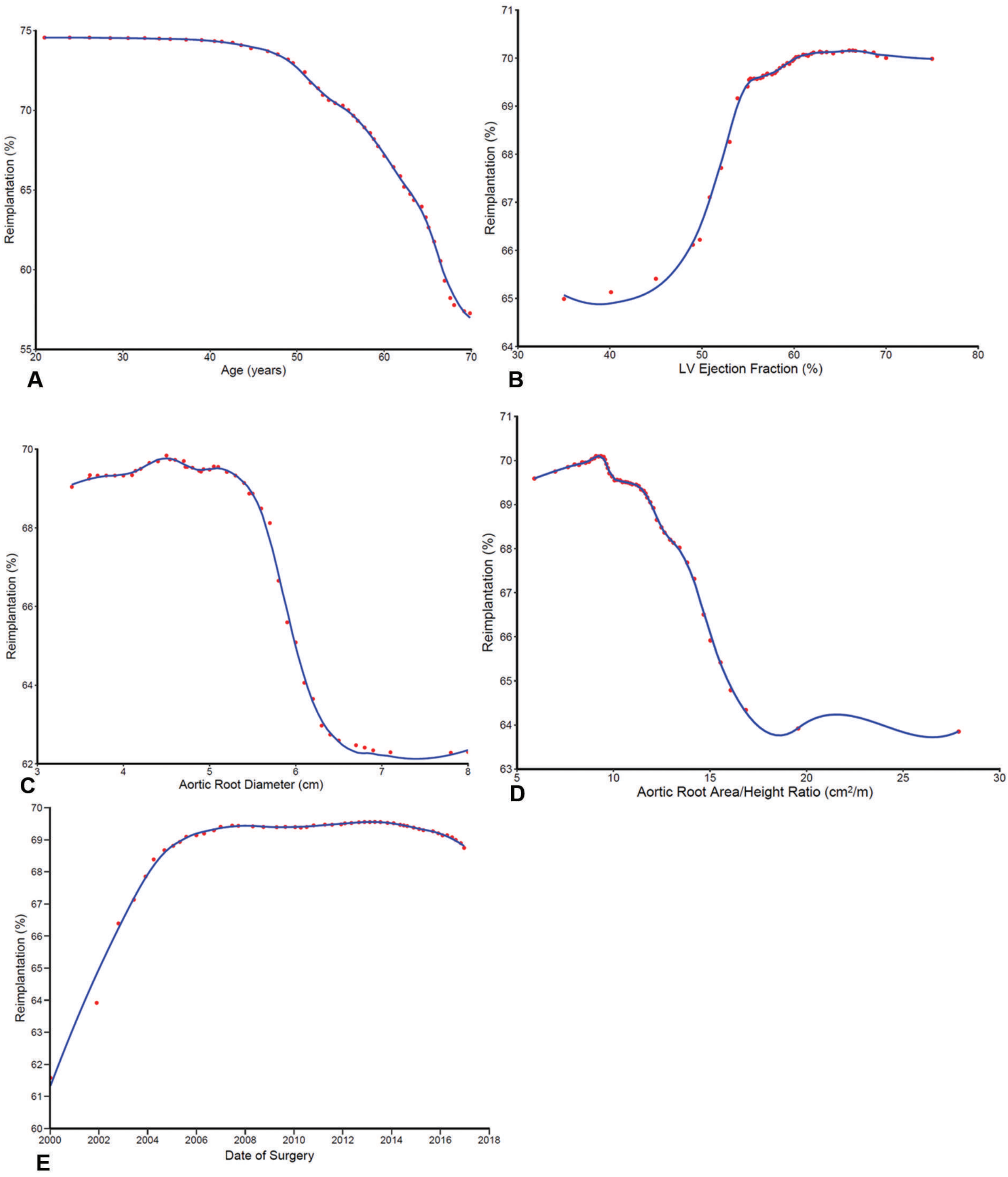

Supplementary Fig. S4 Risk-adjusted probability of reimplantation according to continuous preoperative variables. Each dot is an ensemble average of partial dependence (risk-adjusted) probability based on random forest classification, built using 5000 trees and 50 variables. Solid line is a loess fit. The narrower the horizontal distance between dots, the more patients had values for the continuous variable depicted on the horizontal axis. (**A**) Age. (**B**) Left ventricular (LV) ejection fraction. (**C**) Aortic root diameter. (**D**) Aortic root area/height ratio. (**E**) Date of surgery.

 © 2022. The Author(s).

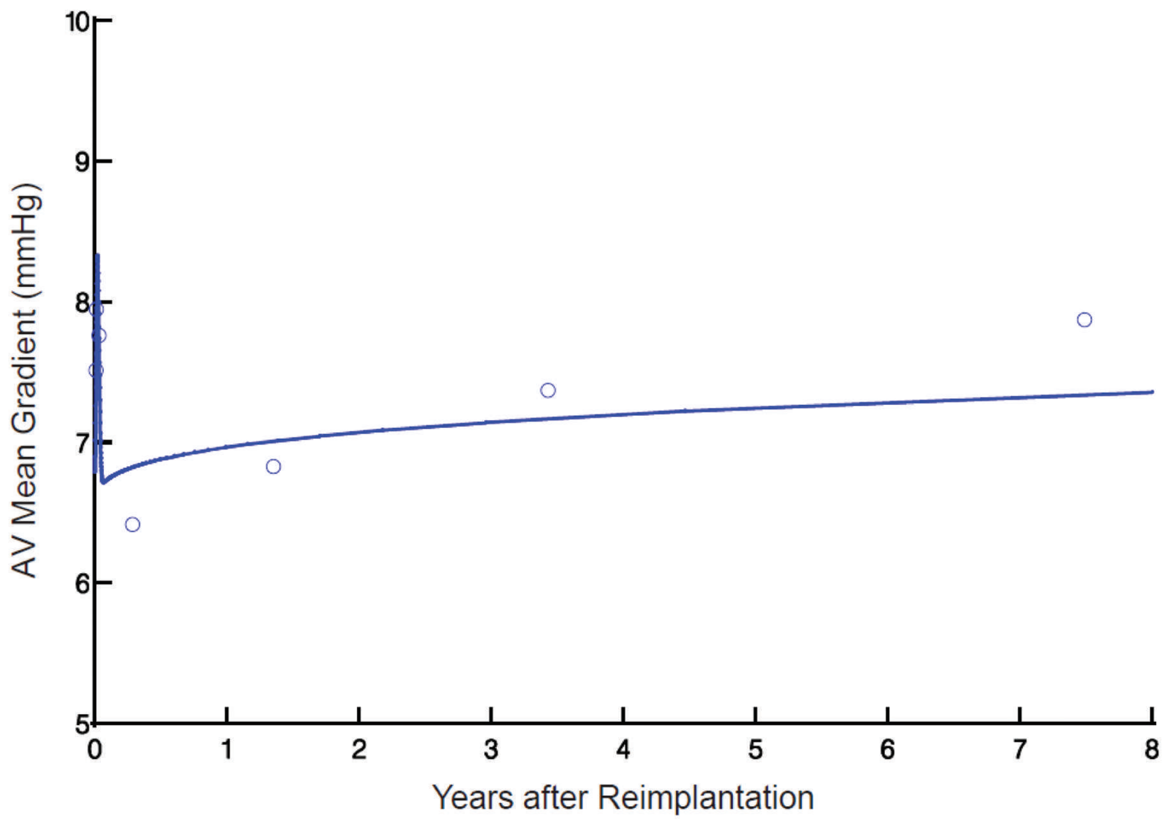

Supplementary Fig. S5 Temporal trend of aortic valve (AV) mean gradient after reimplantation. Solid line represents unadjusted estimates of temporal trend of postoperative AV mean gradient. Symbols represent data grouped (without regard to repeated measurements) within time frames to provide a crude verification of model fit.

 © 2022. The Author(s).

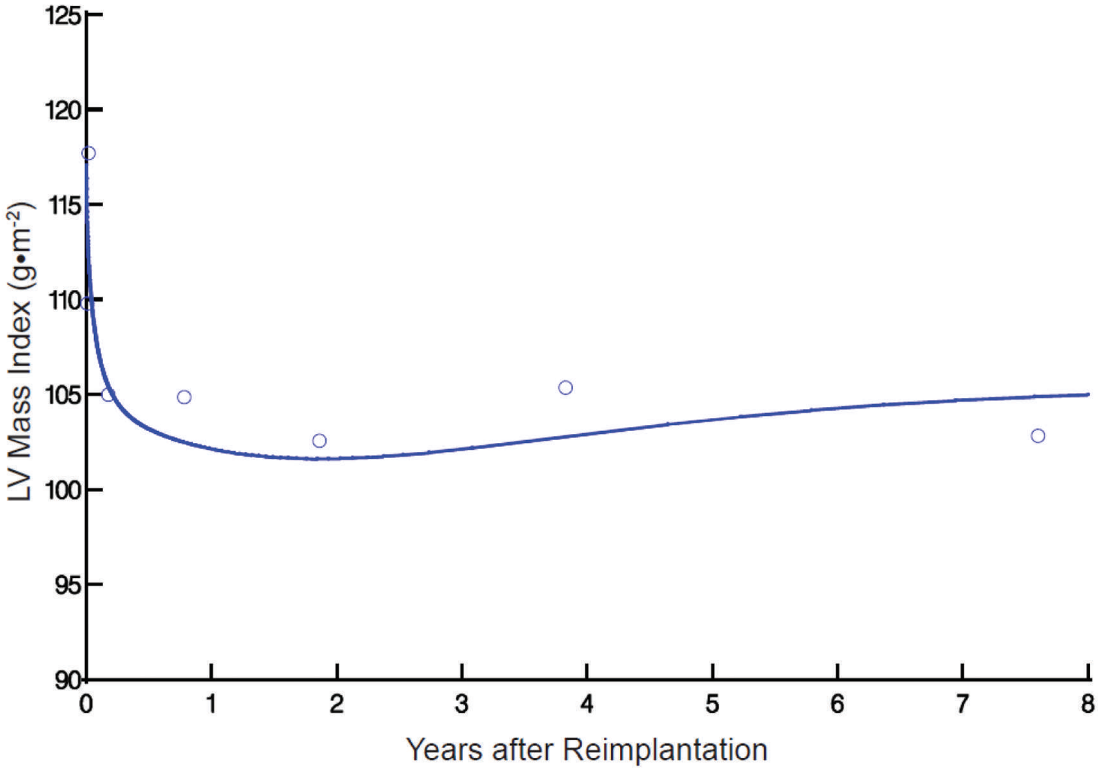

Supplementary Fig. S6 Temporal trend of left ventricular (LV) mass index after reimplantation. Format is as in ►**Supplementary Fig. S5** (online only).

 © 2022. The Author(s).

Supplemental Appendix S1: Variables Considered in the Analysis

Variables associated with Reimplantation versus Bentall Procedure

Demographics
Age (y)*, sex*, race* (white, black, other), height (cm), weight (kg), body mass index (kg•m^{-2})*, body surface area (m^2)*

Symptoms
New York Heart Association functional class (I–IV)*, prior myocardial infarction*

Ventricular Function
Left ventricular (LV) ejection fraction (%)*, fractional shortening

Valve Pathology
Aortic valve regurgitation*, mitral valve regurgitation*, tricuspid valve regurgitation*

Aortic Dimensions
Mid-ascending aorta diameter (cm)*, aortic root diameter (cm)*, aortic root area/height ratio (cm^2/m)*

LV Structure
Diastolic diameter (cm)*, diastolic volume (mL), diastolic volume index (mL•m^{-2}), systolic diameter (cm)*, systolic volume (mL), systolic volume index (mL•m^{-2}), posterior wall thickness (cm), septal wall thickness (cm)*, relative wall thickness*, LV mass (g), LV mass index (g•m^{-2})*

Left Atrial Dimensions
Diameter (cm)*, volume (mL), volume index (mL•m^{-2})

Cardiac Comorbidities
Atrial fibrillation*, heart failure*

Noncardiac Comorbidities
Bilirubin (mg•dL^{-1})*, creatinine (mL•m^{-2}), creatinine clearance (mL•min^{-1})*, glomerular filtration rate (modification of diet in renal disease; mL•min^{-1}•1.73 m^{-2}), blood urea nitrogen (mg•dL^{-1})*, cholesterol (mg•dL^{-1})*, hematocrit (%)*, peripheral arterial disease*, history of hypertension*, treated diabetes*, chronic obstructive pulmonary disease*, history of smoking*, prior stroke*

Coronary Artery Disease
Coronary artery stenosis (none, left main trunk >0% stenosis*, left anterior descending >0% stenosis*, left circumflex >0% stenosis*, right coronary artery >0% stenosis*)

Concomitant Procedures
Coronary artery bypass grafting*, mitral valve procedure*, ablation procedure for atrial fibrillation*

Experience
Date of surgery (years from 1/2000)*

*Variables included in the propensity model.

 © 2022. The Author(s).
